# Seeing it in others versus doing it yourself: Social desirability judgements and conversation production data from autistic and non-autistic children

**DOI:** 10.1177/13623613241292172

**Published:** 2024-11-04

**Authors:** Lauren McGuinness, Kirsten Abbot-Smith, Chiara Gambi

**Affiliations:** 1 University of Kent, UK; 2 University of Warwick, UK

**Keywords:** autism, children, conversation, first impressions, off-topic, response latency, social desirability

## Abstract

On average, groups of autistic individuals are more likely than groups of non-autistic individuals to exhibit unconventional conversational behaviours. We examined autistic and non-autistic children’s social impressions of unconventional responding, as well as actual conversational behaviours in the same participants. Across two studies, 36 autistic and 36 non-autistic matched 9–13-year-olds listened to conversational vignettes which manipulated the relevance and timing of responses produced by the speaker. They then rated the speaker’s social desirability. We also measured the content and latency of the same children’s conversational responses. Autistic children aligned with their non-autistic peers in indicating that they were less likely to befriend, or enjoy interacting with, a speaker who provided off-topic or delayed responses. However, the same autistic children provided more off-topic, and fewer topic-continuing, conversational responses than their non-autistic counterparts. These findings suggest that displaying unconventional conversational behaviours may act as a barrier to friendship or inclusion for autistic children, even when socialising with other autistic peers.

**Lay abstract:**

During a conversation, on average, autistic individuals are often more likely than non-autistic people to provide an off-topic comment and/or to pause for longer before providing a response. One possible explanation for this is that autistic individuals prefer, or are more tolerant of, unconventional communication styles. To explore this possibility, we investigated whether autistic and non-autistic 9–13-year-olds find off-topic or delayed responding a deterrent to friendship or interaction. Participants listened to scripted conversations and then rated social desirability statements, such as ‘I would enjoy chatting to the [target speaker]’. We also examined the prevalence of these behaviours in children’s own conversational responses. We found that autistic children were just as likely as non-autistic children to dis-prefer unconventional conversational responding. Both groups indicated that they were less likely to want to be friends with the speaker, or to chat with them, when they provided off-topic or delayed responses. However, despite their judgements of others, the same autistic children were more likely to provide off-topic responses themselves than their non-autistic peers, as well as giving fewer on-topic responses which facilitate back-and-forth conversation. Overall, this is problematic for autistic children, as our findings suggest that the tendency to exhibit unconventional conversational behaviours will have negative social consequences, even when interacting with other autistic peers.

Despite expressing a desire to be accepted and included by their peers, autistic children and adolescents report that difficulties with social communication act as a significant barrier to forming and maintaining friendships (Cresswell et al., 2019; Sturrock et al., 2022). Whilst a large proportion of autistic children also meet the criteria for structural language impairment (Kjelgaard & Tager-Flusberg, 2001; Loucas et al., 2008), morphosyntactic and lexical difficulties are not universal. In contrast, the tendency to exhibit pragmatic behaviours which restrict one’s ability to engage in reciprocal conversation is a central component of the diagnostic criteria for autism (Diagnostic and Statistical Manual of Mental Disorders (5th ed., text rev.; *DSM*-V-TR); American Psychiatric Association [APA], 2022). One potential explanation for the prevalence of unconventional conversational behaviours among some autistic individuals is a community preference for, or greater tolerance of, alternative communication styles (Granieri et al., 2020). We investigated this possibility by uniquely examining preferences for the communication of others, and the production of certain conversational behaviours, in the *same* autistic participants.

## Social judgements of autistic individuals

The communication style frequently exhibited by autistic individuals can be negatively perceived by the neurotypical majority, resulting in unwelcoming or socially rejecting behaviours (Mitchell et al., 2021). This was illustrated by Sasson et al. (2017; Study 3) who asked neurotypical adults and adolescents to watch brief videos of autistic and non-autistic children retelling a story, and then to provide first impression ratings of the speakers (without knowledge of their diagnosis). Adults and adolescents rated autistic speakers significantly less favourably, indicating that they were more likely to spend time alone, and less likely to get along with others, than non-autistic controls (Sasson et al., 2017; see also Alkhaldi et al., 2021; Boucher et al., 2023; Grossman, 2015, for similar findings with adult raters).

Younger non-autistic children also display negative first impressions of autistic speakers. Stagg et al. (2022) found that neurotypical 6–9-year-olds indicated a lower desire to befriend, or play with, autistic children than non-autistic children across three different stimulus types: silent videos, audio-only and transcribed speech. Similar findings have also been found among neurotypical 10–11-year-olds (Stagg et al., 2014) and 7–12-year-olds (Harnum et al., 2007). This therefore highlights the potentially negative impact that autistic children’s approach to communication may have on their social relationships with non-autistic peers.

## Broad communication preferences

However, whilst non-autistic individuals may negatively judge the distinct communication style of autistic speakers, other autistic individuals may not form the same social impressions. In fact, verbally fluent autistic people may identify with a distinct sub-culture (Straus, 2013) whereby a unique approach to communication has been argued to support positive peer-to-peer interactions between autistic individuals (Heasman & Gillespie, 2019). These communicative preferences may therefore contribute to the use of unconventional conversational behaviours. However, it is not clear whether all autistic adults demonstrate these preferences. Moreover, we do not know when, or if, autistic children may begin to identify with this.

There is also mixed and limited evidence on how the social judgements of autistic individuals may align with, or differ from, that of their non-autistic peers. For example, DeBrabander et al. (2019) found that, while both non-autistic and autistic adults gave similar ratings of personal characteristics, such as assertiveness and intelligence, only autistic raters exhibited no effect of these traits on their subsequent desire for future interactions with autistic speakers. This study also found that disclosing the diagnostic status of the speakers improved non-autistic raters’ social impressions of autistic speakers, but did not affect the judgements of autistic raters (DeBrabander et al., 2019). Using a similar methodology, Grossman et al. (2019) asked autistic and non-autistic adolescents – who were unaware of the speaker’s diagnosis – to watch videos of autistic and non-autistic speakers re-telling a story, and then provide social impression ratings. Both participant groups rated autistic speakers significantly more negatively than non-autistic speakers (e.g. indicating a lower willingness to start a conversation with the speaker). Interestingly, autistic participants provided more negative judgements of traits, such as ‘How likely is it that this person is socially awkward?’, than their non-autistic counterparts. However, both studies used video stimuli, meaning that participants may have based their ratings on several features, including non-verbal behaviours, such as gestures or eye-gaze. It is therefore difficult to isolate which factors were driving the preferences of autistic participants. In addition, in both studies, the target was alone – monologuing to the camera – so it is also unclear how participants may judge speakers in an interactional context.

## Specific conversational behaviours

The aforementioned studies examined participants’ judgements of social communication quite broadly. However, it is particularly important to consider how the impressions of autistic and non-autistic individuals may be shaped by specific, verbal conversational behaviours, as these features are essential for maintaining a conversation and facilitating a smooth interaction. Crucially, verbal communication is central to social engagement during early adolescence, such as through gossiping with peers, which can subsequently impact a child’s friendships or sociometric status (Wargo Aikins et al., 2017).

To focus on specific verbal behaviours, Geelhand et al. (2021) asked autistic and non-autistic adults to make judgements about a range of characteristics from audio recordings of conversations, including the appropriateness of the content, timing and length of responses. Autistic participants were as sensitive as neurotypical controls in detecting features of discourse style and structure, such as relevance and coherence. Participant groups also did not differ in providing less favourable judgements of autistic speakers’ discourse competence (Geelhand et al., 2021).

In contrast, findings from Ying Sng et al. (2020) suggest that autistic adults might be less likely than their non-autistic counterparts to judge unconventional conversational behaviours unfavourably. In this study, autistic and non-autistic participants reported on their experiences of social conversations with an autistic individual. While autistic and non-autistic respondents were equally likely to report unconventional conversational behaviours exhibited by autistic conversation partners (e.g. ‘Starts conversations abruptly’), the autistic participants indicated that these behaviours were less problematic compared to non-autistic raters (Ying Sng et al., 2020). Overall, this demonstrates that there is mixed and limited evidence on how specific discourse features may differentially impact the social impressions of autistic and non-autistic adults.

## Topic management and response timing

There is even less research on how children’s judgements may be shaped by certain conversational behaviours. One specific behaviour that is essential for social conversation is topic management – the ability to introduce relevant topics of shared interest and to develop discourse by providing contingent responses (Tager-Flusberg & Anderson, 1991). The capacity to provide contingent responses – which share the topic of the preceding utterance and provide appropriate information for one’s conversation partner to ‘follow-in’ on (Nadig et al., 2010) – can be associated with a child’s sociometric status within the peer group. For example, Hazen and Black (1989) found that 4–5-year-old children who were disliked by their peers, were less likely to provide on-topic responses during conversations than their well-liked counterparts. Similarly, when Place and Becker (1991) asked neurotypical 9-year-olds to listen to audio-recordings of a child actor providing inappropriate conversational responses, they found that participants were significantly less likely to judge the speaker as likeable, popular or academically skilled when they responded in a delayed or off-topic manner. This therefore highlights how both response timing and topic maintenance are socially significant conversational behaviours among children.

This may be problematic because empirical evidence suggests that autistic children may differ from their non-autistic peers in both areas. That is, difficulties with topic maintenance have been identified among many autistic individuals (see Ying Sng et al., 2018 for a review). More specifically, autistic children have been found to provide less frequent relevant responses about an established conversational topic (Capps et al., 1998), and to exhibit more unannounced shifts to new topics (Bauminger-Zviely et al., 2014; Paul et al., 2009), than other groups of children (see also Tager-Flusberg & Anderson, 1991). However, some studies have not observed these group differences. For example, Nadig et al. (2010) found that the proportion of contingent responses provided by autistic children during conversations with an experimenter was only marginally lower than that of the typically-developing group. Overall, whilst findings are mixed, most studies report that, on average, autistic children are less topic-relevant than their non-autistic peers.

In addition to generating relevant conversational responses, speakers must also determine a suitable time to respond. There is remarkable uniformity across cultures and languages in the response latencies of neurotypical adults, with an average inter-turn gap of just ~200 ms (Stivers & et al, 2009). However, autistic adults have been found to exhibit significantly longer turn-taking gaps than non-autistic adults. For example, Ochi et al. (2019) found the log mean of turn-taking gaps from autistic participants to be almost three-times that of non-autistic adults. However, less is known about group differences among children. While the response latencies of neurotypical children are often much longer than that of adults (over 1 s; Nguyen et al., 2022), there are mixed and limited findings on the response latencies of autistic children. During adult–child interactions, Warlaumont et al. (2010) found no difference in the response latencies of autistic and non-autistic children aged 16–48 months. In contrast, Heeman et al. (2010) found that autistic 4–8-year-olds took significantly longer to respond to questions than non-autistic children, but that groups did not differ when responding to statements. Interestingly, both McKernan et al. (2022) and Parish-Morris et al. (2016) reported a positive association between children’s response latencies and their Autism Diagnostic Observation Schedule (ADOS; Lord et al., 2012) severity scores. As such, while there are some conflicting findings, previous research suggests that, in some contexts, autistic children may take longer to respond to their conversation partner than their non-autistic peers, particularly those with stronger autistic traits.

Overall, a tendency to display these unconventional conversational behaviours – off-topic or delayed responding – may contribute to negative judgements of autistic children from their non-autistic peers (Place & Becker, 1991). However, it is not clear how the social impressions of autistic children might be influenced by these behaviours. Moreover, no previous study has examined social desirability judgements and the production of specific conversational behaviours in the same participants.

## This study

Do autistic and non-autistic children differ in their social desirability ratings of unconventional responding?

Our first research question examined whether autistic children differ from non-autistic peers in finding unconventional (off-topic or delayed) responding a deterrent to friendship or interaction. This is the first time in the autism field that these two conversational behaviours have been examined in isolation from other factors, such as nonverbal cues, which might colour participants’ judgements.

We recruited 36 autistic and 36 non-autistic 9–13-year-old children. This age range was chosen as it represents a phase in which social conversation starts to become crucial for social interactions and peer engagement (Wargo Aikins et al., 2017), as opposed to conversation around an object of play. Across two studies, participants listened to 30-second vignettes of dyadic conversations between a male and female actor, which were manipulated to investigate the factors of topic relevance and response timing. For each vignette, participants rated social desirability statements about the target speaker, such as ‘I would enjoy chatting to the [target speaker]’.

### Group production differences in the same participants

This study is the first to investigate social desirability judgements and conversational behaviours in the same participants – concurrently examining a child’s preferences for the communication of others and their own conversational ability. As such, our second – and crucial – research question was whether the same autistic children differed from matched non-autistic peers in their own use of these unconventional conversational behaviours (off-topic or delayed responding). This is a fundamental step in unpicking whether there are differences in the conversational styles of autistic and non-autistic children, and if so, how these may map onto their communicative preferences.

## Method

### Participants

In line with our pre-registration (osf.io/j2stz), 36 autistic and 36 non-autistic 9–13-year-olds were tested. Using G-Power, we determined that this sample size would allow us to detect a medium-to-large effect (*d* = 0.7) at 80% power, and a *p* = 0.05 level of significance, with an independent samples *t*-test. None of the participants had diagnosed learning or hearing disabilities. All had heard British English spoken to them since birth and heard no other languages at home. 89% of parents stated that their children were White British, 7% belonged to Mixed or Multiple ethnic groups and 4% identified as Black, Black British, Caribbean or African. According to parental report, non-autistic participants had no difficulties with language, attention or reading, nor any suspected or diagnosed neurodevelopmental difficulties. All autistic children had a formal diagnosis of autism from a paediatrician or clinical psychologist, evidence of which was shown to the experimenter. 25% of autistic participants had a co-morbid diagnosis of attention-deficit hyperactivity disorder (ADHD).

Participants were recruited from local schools, social media and the Kent Child Development Unit database. Of the autistic sample, 56% attended either a ‘special school’ or ‘specialist resource provision’ attached to a mainstream school. Autistic community members were not involved in the development of the reported studies.

Diagnostic groups were matched on chronological age, sex ratio and socioeconomic status. As shown in Table 1, groups also did not significantly differ in their scores on the ‘Recalling Sentences’ sub-test from the Clinical Evaluation of Language Fundamentals^®^ – Fourth Edition (CELF^®^-4, Wiig et al., 2006), nor the ‘Matrices’ sub-test of the Wechsler Abbreviated Scale of Intelligence (WASI-II, Wechsler, 2011). Ethical approval was obtained for this study (University of Kent).

**Table 1.** Means (*SD* in brackets) for participant characteristics.

	Autistic (*n* = 36, 25 males)	Non-autistic (*n* = 36, 25 males)	p	*d*
	Mean (*SD*)	Mean (*SD*)		
Chronological age (Months)	138.78 (17.56)	138.92 (18.74)	0.974	–0.01
Recalling sentences CELF-4 scaled score (Language)	10.33 (3.26)	10.47 (2.36)	0.837	–0.05
WASI matrices sub-test T-score (non-verbal reasoning)	51.83 (8.48)	51.16 (7.67)	0.728	0.08
Maternal education (1–8)^a^	5.06 (2.20)	5.61 (1.99)	0.265	–0.26
Postcode Income Decile (1–10)^b^	6.91 (2.21)	6.11 (2.30)	0.134	0.36
Social Responsiveness Scale T-score^c^	82.97 (8.79)	45.94 (6.99)	< 0.001	4.66

CELF: Clinical Evaluation of Language Fundamentals; WASI: Wechsler Abbreviated Scale of Intelligence.

a In England, Wales and Northern Ireland, formal education qualifications are split into eight levels. Levels 1–3 involve school and college qualifications, while Levels 4–8 reflect university qualifications, from a Higher National Certificate (4) to a Doctorate (8) (Department for Education, 2023).

b The Index of Multiple Deprivation is a measure of relative deprivation in England, where 1 are the most deprived areas and 10 are the least deprived areas (Ministry of Housing, Communities & Local Government, 2019).

c Constantino & Gruber, 2007.

### Overall procedure and design

Across two testing sessions, each child completed tasks while verbally interacting with a female, native English-speaking experimenter (the first author). Since testing commenced under COVID-19 restrictions, 86% of these sessions (for both diagnostic groups) took place via Zoom, whereby parents supplemented online video-recordings with audio-recordings at the participant’s end, to allow for accurate measurement of conversational response latencies. For the 10 children tested in-person, the experimenter sat next to the child in front of the screen, audio-recorded on a Dictaphone, and video-recorded via Zoom.

During these sessions, each child participated in two concurrent studies – both of which examined one ‘unconventional’ conversational behaviour. Study 1 investigated the role of topic relevance and Study 2 investigated the role of response timing. For both studies, we obtained judgement and production data from the same children.

#### Social desirability judgement task

**Figure 1.**
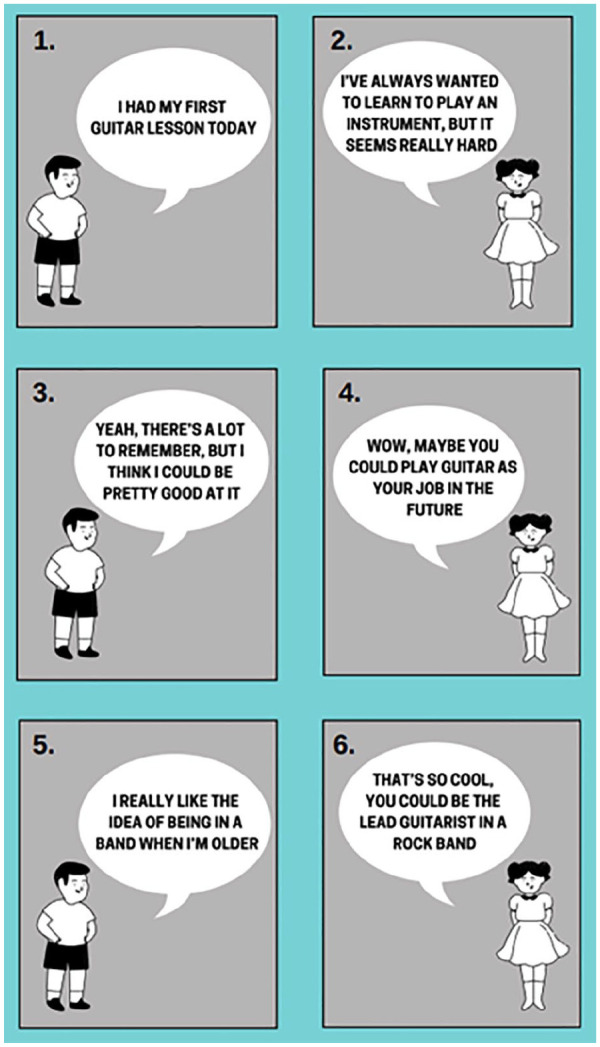
Example comic-strip-style graphic of conversational vignette.

The task was presented as a Qualtrics survey, but the experimenter was always co-present (in-person or virtually). Participants were instructed to provide ratings based on how the target speaker behaved during the conversation, using sliders from ‘No’ (Sad face) to ‘Yes’ (Smiley face). Each six-turn audio-only vignette was presented alongside a comic-strip-style graphic depicting the conversation between two cartoon speakers (see Figure 1 and Supplemental materials).

For each vignette, participants rated four social desirability statements on a 0–100 scale. The first two statements captured participants’ ‘Personal’ preferences, and the second two reflected their understanding of ‘Societal’ preferences:

(a) Personal: ‘I would like to be friends with the [target speaker]’
(b) Personal: ‘I would enjoy chatting with the [target speaker]’
(c) Societal: ‘Most other people would like the [target speaker]’
(d) Societal: ‘Most other people would think the [target speaker] is good at having a conversation’

Participants also verbally justified their judgements. They were asked ‘*Why did you choose those ratings*?’ for each set of sliders.

#### Social desirability judgement design

We did not have predictions regarding an interaction between response relevance and timing. We therefore pre-registered the studies (prior to data collection) such that the effects of topic relevance and response timing would not be examined in the same analyses. Instead, these factors were investigated separately in two concurrent studies. As such, the same ‘conventional responding’ vignettes (On-topic + Typical timing) are the control condition for both Study 1 and 2.

In both studies, we used a 2 (Diagnostic Group: Autistic (ASC) versus Non-Autistic (NA)) × 2 (Condition: Conventional vs Unconventional) × 2 (Preference Type: Personal vs Societal) design, with the latter two factors as within-subjects variables. Six participants from each diagnostic group were assigned to each of the six script orders. Each of the conversation content types (hobbies, holidays, and so on) appeared evenly across the whole sample. Nine vignettes were presented in three blocks of three (On-topic + Typical; On-topic + Lag; Off-topic + Typical). The presentation order within blocks was randomised.

#### Production measure

Between each block of the judgement task, the experimenter (E) elicited naturalistic conversational responses from the participant. Across sessions, E used 15 probes – which were declarative statements somewhat related to something the participant had just seen or heard – as part of the experimental procedure (see Supplemental materials). For example, when the child had just seen a funny video of a dog on the screen, E would say (2):

(2) ‘I had a dog like that when I was little, but he was a bit naughty’

### Study 1: judgements and production of topic relevance

The first study examined how children judged relevance in others, and the extent to which the same children provided relevant conversational responses themselves. Regarding the judgements, participants heard vignettes in two conditions: On- versus Off-topic. In three control vignettes, the target speaker provided On-topic responses, and in three other vignettes the target speaker provided Off-topic responses which were irrelevant to the prior utterance, as in (3) below. All responses in Study 1 had Typical timing (200 ms from the offset of prior the utterance).

(3) A: ‘I went to that new restaurant in town last night’.
  B: ‘Oh no, I think my library books are due in today’.

Regarding production, the children’s responses to conversation probes, such as (2), were coded for contingency, following a similar criterion to Pagmar et al. (2022). Participants’ responses were coded as either contingent, non-contingent, minimal or non-verbal (see Table 2). To check reliability, 15% of responses to probes were double-coded by a second rater. This demonstrated near-perfect agreement (κ = 0.94).

**Table 2.** Coding of responses to probes: Categories, definitions and examples.

Category	Definition	Example responses provided to probe (2)
Contingent	Statements or questions which are appropriate, relevant and add information to the probe (see Bloom et al., 1976).	‘*My friend had a dog like that*’ (PA12)
Non-contingent	Responses which do not maintain the topic of the probe, such as switches to talking about the environment, returns to previous topics, and utterances which are only tangentially related to the probe.	‘*Yeah, I know these lyrics [starts singing]*’ (PA9)
Minimal	Responses which are not off-topic, but do not add information to the probe (Pagmar et al., 2022). These include short one-or two-word comments or affective phrases.	‘*That’s cute*’ (PN8) ‘*[Laughs] Okay*’ (PA19) ‘*Mmm*’ (PA11)
Non-verbal	A non-verbal behaviour provided in the absence of a verbal response to a probe within 3000 ms.	[Smiles] (PN6) [Stares ahead] (PA6)

### Study 2: judgements and production of response timing

The second study examined how children judged response timing in others, as well as the response latencies of the same children during conversation. Regarding the judgements, participants heard vignettes in two conditions: Typical versus Delayed timing. In three control vignettes, the target speaker responded after a Typical amount of time – 200 ms after the offset of the first speaker’s utterances. In the Delayed condition (three vignettes), the target speaker responded 3000 ms after the offset of the first speaker’s utterances. All responses in Study 2 were On-topic. Regarding production, the children’s response latencies were measured using Audacity from the offset of the 15 experimenter probes, such as (2) above.

## Study 1 results

We pre-registered our analysis plan (https://osf.io/j2stz/). For each participant, we computed a personal preference rating for each vignette by conflating across statements ‘(a)’ and ‘(b)’ above (which were highly correlated *r*(646) = 0.87, *p* < 0.001). We also calculated a societal preference rating by conflating across statements ‘(c)’ and ‘(d)’ above (which were also highly correlated *r*(646) = 0.88, *p* < 0.001).

### Do autistic and non-autistic children differ in their social desirability ratings of unconventional responding?

**Figure 2.**
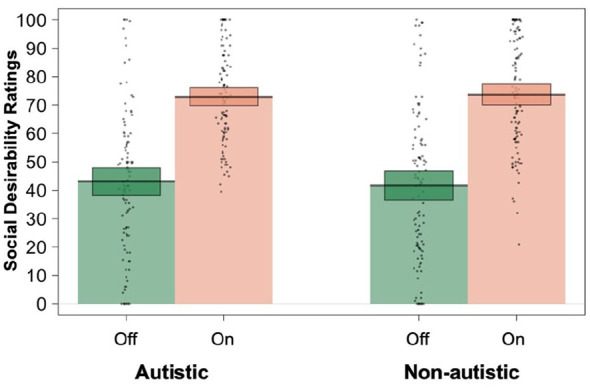
Autistic and non-autistic participants’ ratings of on- versus off-topic responses.

A linear mixed effects model revealed a significant main effect of Relevance on participants’ social desirability ratings (*B* *=* –32.13, *SE* = 3.34, χ^2^(1) = 22.28, *p* < 0.001). On-topic responses (*M* = 73.89) were rated more favourably than Off-topic responses (*M* = 41.76). However, there was no main effect of Diagnostic Group (*B* *=* 0.21, *SE* = 3.07, χ^2^(1) = 0.00, *p* = 0.945).^1^ Autistic and non-autistic participants did not significantly differ in their mean ratings of On-topic (ASC = 73.13 vs NA = 74.65) or Off-topic (ASC = 42.31 vs NA = 41.21) vignettes (see Figure 2). There was also no main effect of Preference Type (*B* *=* –0.03, *SE* = 0.92, χ^2^(1) = 0.00, *p* = 0.976), with participants providing similar personal (*M* = 57.84) and societal ratings (*M* = 57.81). None of the interaction effects were significant (see Table 3).

**Table 3.** Interaction effects for Relevance x Diagnostic Group x Preference Type analysis (Model 1).

	*B*	*SE*	χ^2^	*p*
Relevance × Diagnostic Group	–2.61	5.62	0.22	0.639
Relevance × Preference Type	–2.33	1.84	1.61	0.204
Preference Type × Diagnostic Group	1.10	1.84	0.36	0.548
Relevance × Preference Type × Diagnostic Group	–0.80	3.68	0.05	0.828

Participants’ chronological age significantly moderated the relationship between the Relevance manipulation and their social desirability ratings (*B* = 0.17, *SE* = 0.07, *p* < 0.05). Older children provided less favourable ratings of Off-topic responses (*EMM* = 38.3) than younger children (*EMM* = 45.2). There was no moderation effect for participants’ scaled scores on the CELF Recalling Sentences subtest (*B* = 0.06, *SE* = 0.50, *p* = 0.905).

A content analysis of participants’ verbal justifications of their Personal ratings for Off-topic vignettes revealed that 70% of comments contained a spontaneous reference to the relevance of the speaker’s responses (ASC: 73% vs NA: 68%; see Supplemental materials for coding framework). This included comments such as ‘*she was changing the subject often*’ (N29) and ‘*she was a bit off-topic*’ (A6). Across groups, 14% of justifications referenced active listening, such as ‘*She’s not listening to what the boy’s saying’* (A30), and 3% mentioned possible difficulties experienced by the target speaker, such as ‘*She might be a bit confused and have like a disability*’ (A3).

### Group production differences in the same participants

Following our pre-registered plan for the production data (https://osf.io/26djt/), we focused on the analysis of, first, contingent responses (as opposed to non-contingent, minimal or non-verbal responses) and second, non-contingent responses (as opposed to contingent, minimal or non-verbal responses). For both analyses, we ran logistic linear mixed effects models, with diagnostic group (ASC coded as 0.5, NT as –0.5) as a contrast-coded fixed effect, and both participants and items (conversation probes) included as random intercepts.

For both analyses, Diagnostic Group was a significant predictor. Autistic participants exhibited significantly lower rates of contingent responding to probes than did non-autistic participants (see Table 4; *B* *=* 0.86, *SE* = 0.33, χ^2^(1) = 6.75, *p* < 0.01). Despite rating off-topic responding unfavourably in others, autistic participants also exhibited significantly higher rates of non-contingent responding than non-autistic participants (see Table 4; *B* *=* –1.41,*SE* = 0.53,χ^2^(1) = 7.04, *p* < 0.01).

**Table 4.** Mean proportions of responses provided by each diagnostic group.

Response	Autistic *M* proportion	Non-autistic *M* proportion
Contingent	0.40	0.57
Non-contingent	0.09	0.03
Minimal	0.37	0.29
Non-verbal	0.14	0.11

We also investigated whether participants’ mean social desirability ratings of On- or Off-topic vignettes correlated with their own production of contingent or non-contingent responses. We examined potential relationships in the whole sample, and separately in each diagnostic group. Only the negative relationship between participants’ mean ratings of Off-topic vignettes and the mean proportion of contingent responses produced reached significance, and only across the whole sample (*r*_s_ = –0.24, *p* < 0.05). In other words, participants who provided a higher proportion of contingent responses also tended to judge Off-topic vignettes more unfavourably. None of the other correlations were significant (all *r*_s_ < 0.24, all *p* > 0.148; see Supplemental materials).

## Study 2 results

### Do autistic and non-autistic children differ in their social desirability ratings of unconventional responding?

Here, we found a similar pattern of results as in Study 1. A linear mixed effects model revealed a significant main effect of Timing on participants’ social desirability ratings (*B* *=* –11.66, *SE* = 1.82, χ^2^(1) = 14.21, *p* < 0.001). Responses provided after a Typical amount of time (*M* = 73.89) were rated more favourably than Delayed responses (*M* = 62.23). Again, there was no main effect of Diagnostic Group (*B* *=* 3.92, *SE* = 3.68, χ^2^(1) = 1.16, *p* = 0.281).^2^ Autistic and non-autistic participants did not significantly differ in their mean ratings of Typical (ASC = 73.13 vs NA = 74.65) or Delayed (ASC = 59.07 vs NA = 65.40) responses. There was also no main effect of Preference Type (*B* *=* –0.34, *SE* = 1.20, χ^2^(1) = 0.08, *p* = 0.775), with participants providing similar personal (*M* = 68.23) and societal ratings (*M* = 67.89). None of the interaction effects were significant (see Table 5).

**Table 5.** Interaction effects for Timing x Diagnostic Group x Preference Type analysis (Model 2).

Interaction	*B*	*SE*	χ^2^	*p*
Timing × Diagnostic Group	4.82	4.06	1.43	0.231
Timing × Preference Type	–2.96	1.70	3.03	0.082
Preference Type × Diagnostic Group	3.20	2.39	1.82	0.178
Timing × Preference Type × Diagnostic Group	3.40	3.40	1.00	0.317

Participants’ social desirability ratings were not significantly moderated by their age (*B* = –0.08, *SE* = 0.08, *p* = 0.317). However, there was a marginal effect of language ability (*B* = 1.07, *SE* = 0.56, *p* = 0.058). Participants with higher scaled scores on the CELF Recalling Sentences subtest provided less favourable ratings of Delayed responses (*EMM* = 59.5) than those with lower scaled scores (*EMM* = 66.8).

A content analysis of participants’ verbal justifications of their Personal ratings of Delayed vignettes revealed that 45% of comments contained a spontaneous reference to the timing of the speaker’s responses (ASC: 54% vs NA: 35%). This included comments such as ‘*There’s a really awkward pause*’ (A3) and ‘*She waited quite a long time to reply*’ (N20). Across groups, 9% of justifications referenced active listening, such as ‘*She wasn’t listening or wasn’t interested*’ (A25), while 5% included possible difficulties experienced by the target speaker, such as ‘*She doesn’t know what to say*’ (A16).

### Group production differences in the same participants

**Figure 3.**
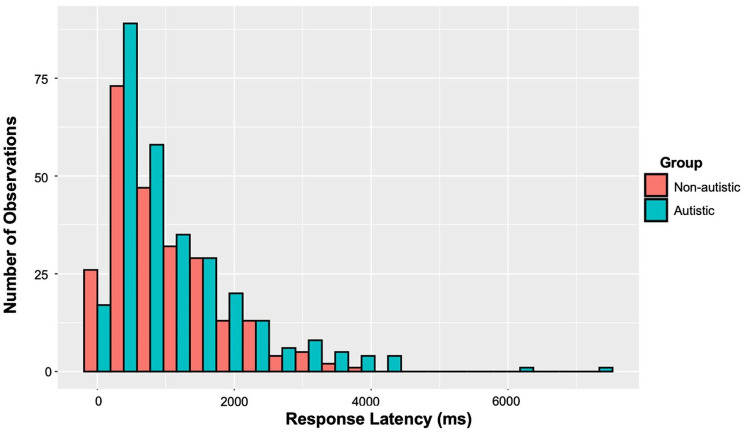
Distribution of response latencies.

Due to technical difficulties with parental recordings, the response latencies of 25% of the sample could not be measured. The following analysis is, therefore, based on 30 autistic and 24 non-autistic participants. These data were not normally distributed, with a long right-tail (see Figure 3). A Bayesian lognormal model was run to examine group differences in the central tendency and variability of response latencies. We ran four chains for 4000 iterations, with a warm-up period of 2000 iterations and the default brms (non-informative) priors. We report an estimate (*B*), estimated error (EE) and the 95% credible interval (CrI) for both parameters. Here, the presence of zero in the 95% CrI would suggest that there is not sufficient evidence that the estimate is different from zero.

The hypothesis that groups differed was not supported by the model. Autistic and non-autistic participants’ response latencies did not significantly differ on the measure of central tendency (*B* = –0.18, EE = 0.16, 95% CrI = [–0.50, 0.14]) nor variability (*B* = 0.19, EE = 0.12, 95% CrI = [–0.04, 0.42]).^3^

We also investigated whether participants’ mean social desirability ratings of Delayed vignettes correlated with their own mean response latencies. Across all participants, we found no significant correlation (*r*_s_ = 0.06, *p* = 0.657). This was also the case when examining this relationship separately in each diagnostic group (see Supplemental materials).

## Discussion

These studies are the first to concurrently investigate autistic and non-autistic children’s social desirability judgements of unconventional responding, and group differences in the production of the same specific conversational behaviours. To examine social impressions of off-topic and delayed responding, autistic and non-autistic 9–13-year-olds listened to 30-second vignettes of dyadic conversations, which were manipulated to investigate the factors of topic relevance and response timing. Participants then rated social desirability statements about the target speaker. We also investigated whether the same autistic children differed from well-matched non-autistic peers in their own use of these unconventional behaviours (off-topic or delayed responding) during conversations with the experimenter.

Our findings demonstrated that, in late childhood and early adolescence, verbally fluent autistic children are just as likely as their non-autistic peers to dis-prefer social interaction with speakers who provide off-topic or delayed conversational responses. However, despite their preferences regarding the communication of others, the same autistic children produced significantly more non-contingent responses, and significantly fewer contingent responses, than their non-autistic counterparts. Groups did not significantly differ in the duration or variability of their response latencies.

### Social consequences

These results suggest that exhibiting unconventional conversational behaviours will have significant social consequences, since participants indicated that they were less likely to want to be friends with, or to interact with, someone who responds in an off-topic or delayed manner. This is particularly problematic for autistic children who, on average, provided significantly more off-topic responses than non-autistic participants. As such, negative first impressions based on conversational style may act as a barrier to the peer acceptance and inclusion desired by many autistic children and adolescents (Cresswell et al., 2019). This also reflects the self-reports of autistic children, who state that social communication difficulties can preclude them from peer relationships (Sturrock et al., 2022). Overall, this may also contribute to other adverse experiences of some autistic youths, including elevated rates of bullying (Maïano et al., 2015) and loneliness (Hymas et al., 2022), as well as frequent co-occurring anxiety (van Steensel et al., 2011).

Importantly, this is the case even if autistic children only socialise among their ‘own’ community, such as with other autistic children in a specialist educational provision. Of our autistic sample, 56% of participants attended either a special school or a specialist resource provision attached to a mainstream school, for which they could only qualify if they require a high level of specialist support (Kent County Council, 2023). Previous research has identified unique dimensions of neurodivergent interactions which can facilitate rapport between autistic individuals, such as generous assumptions of common ground (Heasman & Gillespie, 2019) and reduced reliance on mutual gaze or backchannelling (Rifai et al., 2022). However, our findings suggest that negative perceptions of two other conversational behaviours – off-topic and delayed responding – would instead act as a barrier to social inclusion in peer-to-peer interactions between autistic children.

That said, it is possible that as autistic children grow older, they might become more tolerant of unconventional conversational behaviours. This notion aligns with previous research, which has found that autistic adults judge unconventional communication styles as less problematic (Ying Sng et al., 2020), and less of a barrier to future interactions (DeBrabander et al., 2019), than non-autistic adults.

### What is driving the conversational behaviours of autistic children?

By examining the timing and relevance of participants’ conversational responses, our studies also contribute to the existing literature on the communication style of autistic children. In particular, our results add to the limited and mixed evidence regarding autistic and non-autistic children’s response latencies. That is, group differences did not reach significance, and there was high variability in both groups (see Figure 3). However, across all participants and probes, 29 responses could be classified as Delayed, with latencies in excess of 3000 ms. Of these, 76% were from autistic participants. Future studies with a larger sample size are needed to ascertain whether this is indicative of a subgroup within the autistic population.

Our findings of higher rates of non-contingent responding, and lower rates of contingent responding, in autism also mirror the majority of previous studies (see Ying Sng et al., 2018 for a review). However, it is important to note that, on average, non-contingent responses made up less than 10% of all responses provided by autistic children. Future studies could further develop our social desirability paradigm to investigate whether a 10% frequency of off-topic responding is indeed sufficient to impact a child’s social desirability. Furthermore, given that minimal or non-verbal responses were much more frequently produced, future research is needed to examine the relative social desirability of these other conversational response types.

Crucially, we found no evidence of a distinct preference for unconventional conversational behaviours among autistic children which might have contributed to the higher rate of off-topic responding. The absence of significant correlations between autistic participants’ preferences for topic relevance in others and their own conversational behaviour suggests a potential disconnect. One possible explanation for this is reduced self-awareness. Previous research suggests that some autistic individuals may struggle with psychological self-awareness (Williams, 2010). More specifically, Johnson et al. (2009) found evidence of diminished self-awareness of autistic traits among autistic children and adolescents when comparing self-reports and parent reports. As such, it is possible that autistic children may be unaware of their own conversational traits, meaning that they do not apply their communicative preferences to their own behaviour.

A similar pattern has also been observed in some children with social communication disorder (Lockton et al., 2016), whereby participants exhibited explicit knowledge of pragmatic rules, but did not apply them to their own conversational responses. Given the impact that displaying unconventional conversational behaviours could have on autistic children’s peer relationships, future studies need to explore the degree to which those who display these conversational traits are aware of their own behaviour.

### Limitations

One possible limitation of this research is our use of vignettes which were ‘enacted’ by child actors, as these stimuli are not as ecologically valid as real-world conversations. However, it is important to note that this was necessary to provide the rigorous experimental control required to isolate and identify specific conversational behaviours that influence participants’ social judgements.

In addition, our findings may not be generalisable to the autistic population as a whole. Since we closely matched our two diagnostic groups to rule out intellectual or language impairments as an alternative explanation for atypical conversational behaviours, we did not test any autistic individuals with moderate or severe intellectual disabilities or language impairments.

Moreover, our sample was also not culturally diverse, since all participants live within a certain county in Southern England, which is predominantly White British. There are likely to be cross-cultural differences in many aspects of social communication (Gabbatore & et al, 2023), which impact not only child conversational behaviours, but also their social judgements of these. That said, a preference for relevance has been argued to be universal (Grice, 1975; Sperber & Wilson, 1995), and cross-linguistic studies of conversational response timing have revealed a surprising uniformity (Stivers & et al, 2009). Nonetheless, future studies should explore these communicative behaviours and preferences from both autistic and non-autistic children growing up in a variety of non-WEIRD cultures (Nielsen et al., 2017).

## Conclusion

Across two studies, autistic children aligned with their non-autistic peers in indicating that they were less likely to befriend, or enjoy interacting with, a speaker who provided off-topic or delayed conversational responses. However, during conversations with the experimenter, the same autistic children were found to provide more off-topic, and fewer topic-continuing, conversational responses than their non-autistic counterparts. More research is needed to establish whether the apparent disconnect between autistic children’s preferences for topic relevance in others, and their own conversational production, could be attributed to reduced self-awareness. Regardless, our findings illustrate how displaying unconventional conversational behaviours may negatively impact the social experiences of autistic children, even when interacting with other autistic peers.

## Supplemental Material

sj-docx-1-aut-10.1177_13623613241292172 – Supplemental material for Seeing it in others versus doing it yourself: Social desirability judgements and conversation production data from autistic and non-autistic children Supplemental material, sj-docx-1-aut-10.1177_13623613241292172 for Seeing it in others versus doing it yourself: Social desirability judgements and conversation production data from autistic and non-autistic children by Lauren McGuinness, Kirsten Abbot-Smith and Chiara Gambi in Autism

## Notes

1. There was also no main effect of Diagnostic Group when autistic participants with co-morbid ADHD were excluded from analyses (*B* *=* –0.78, *SE* = 3.45, χ^2^(1) = 0.05, *p* = 0.818). There was also no main effect of Diagnostic Group when the autistic sample only included participants who attended specialist schooling (*B* *=* 3.35, *SE* = 3.58, χ^2^(1) = 0.90, *p* = 0.342).

2. There was also no main effect of Diagnostic Group when autistic participants with co-morbid ADHD were excluded from analyses (*B* *=* 2.59, *SE* = 3.74, χ^2^(1) = 0.49, *p* = 0.482). There was also no main effect of Diagnostic Group when the autistic sample only included participants who attended specialist schooling (*B* *=* 6.61, *SE* = 4.33, χ^2^(1) = 2.35, *p* = 0.125).

3. A non-parametric test was also conducted to compare the mean latencies of autistic (*M* = 1185.16 ms) and non-autistic (*M* = 937.45 ms) participants. This revealed only a marginally significant difference (*W* = 388, *p* = 0.090).

## References

[bibr1-13623613241292172] Alkhaldi R. S. Sheppard E. Burdett E. Mitchell P. (2021). Do neurotypical people like or dislike autistic people? Autism Adulthood, 3(3), 275–279. https://doi.or/10.1089/aut.2020.0059 36605364 10.1089/aut.2020.0059 PMC8992906

[bibr2-13623613241292172] American Psychiatric Association. (2022). Diagnostic and statistical manual of mental disorders (5th ed., text rev.). 10.1176/appi.books.9780890425787

[bibr3-13623613241292172] Bauminger-Zviely N. Karin E. Kimhi Y. Agam-Ben-Artzi G. (2014). Spontaneous peer conversation in preschoolers with high-functioning autism spectrum disorder versus typical development. Journal of Child Psychology and Psychiatry, 55(4), 363–373. https://doi.org/10.1111.jcpp.12158 24304222 10.1111/jcpp.12158

[bibr4-13623613241292172] Bloom L. Rocissano L. Hood L. (1976). Adult-child discourse: Developmental interaction between information processing and linguistic knowledge. Cognitive Psychology, 8(4), 521–552. 10.1016/0010-0285(76)90017-7

[bibr5-13623613241292172] Boucher T. G. Lukacs J. N. Schreer N. E. Iarocci G. (2023). Negative first impression judgements of autistic children by non autistic adults. Frontiers in Psychiatry, 14, 1–13. 10.3389/fpsyt.2023.1241584 PMC10587469 37867780

[bibr6-13623613241292172] Capps K. Kehres J. Sigman M. (1998). Conversational abilities among children with autism and children with developmental delays. Autism, 2(4), 325–344. 10.1177/1362361398024002

[bibr7-13623613241292172] Constantino J. N. Gruber C. P. (2007). Social Responsiveness Scale (SRS). Western Psychological Services.

[bibr8-13623613241292172] Cresswell L. Hinch R. Cage E. (2019). The experiences of peer relationships amongst autistic adolescents: A systematic review of the qualitative evidence. Research in Autism Spectrum Disorders, 61, 45–60. 10.1016/j.rasd.2019.01.003

[bibr9-13623613241292172] DeBrabander K. M. Morrison K. E. Jones D. R. Faso D. J. Chmielewski M. Sasson N. J. (2019). Do first impressions of autistic adults differ between autistic and nonautistic observers? Autism in Adulthood, 1(4), 250–257. 10.1089/aut.2019.0018 36601322 PMC8992824

[bibr10-13623613241292172] Department for Education. (2023). What qualification levels mean. https://www.gov.uk/what-different-qualification-levels-mean/list-of-qualification-levels

[bibr11-13623613241292172] Gabbatore I. Dindar K. Pirinen V. Vähänikkilä H. Mämmelä L. Kotila A. Bosco F. M. Leinonen E. Loukusa S. (2023). Silent Finns and talkative Italians? An investigation of communicative differences and similarities as perceived by parents in typically developing children. First Language, 43(3), 313–335. 10.1177/01427237221149310

[bibr12-13623613241292172] Geelhand P. Papastamou F. Deliens G. Kissine M. (2021). Judgments of spoken discourse and impression formation of neurotypical and autistic adults. Research in Autism Spectrum Disorders, 82, 101742. 10.1016/j.rasd.2021.101742

[bibr13-13623613241292172] Granieri J. E. McNair M. L. Gerber A. H. Reifler R. F. Lerner M. D. (2020). Atypical social communication is associated with positive initial impressions among peers with autism spectrum disorder. Autism, 24(7), 1841–1848. 10.1177/1362361320924906 32498545

[bibr14-13623613241292172] Grice H. P. (1975). Logic and conversation. In Cole P. Morgan J. L. (Eds.), Syntax and semantics 3: Speech acts (pp. 41–58). Academic Press.

[bibr15-13623613241292172] Grossman R. B. (2015). Judgments of social awkwardness from brief exposure to children with and without high-functioning autism. Autism, 19(5), 580–587. 10.1177/1362361314536937 24923894 PMC4485991

[bibr16-13623613241292172] Grossman R. B. Mertens J. Zane E. (2019). Perceptions of self and other: Social judgments and gaze patterns to videos of adolescents with and without ASD. Autism: The International Journal of Research and Practice, 23(4), 846–857. 10.1177/1362361318788071 30014714 PMC6403013

[bibr17-13623613241292172] Harnum M. Duffy J. Ferguson D. A. (2007). Adults’ versus children’s perceptions of a child with autism or attention deficit hyperactivity disorder. Journal of Autism and Developmental Disorders, 37, 1337–1343. 10.1007/s10803-006-0273-0 17080272

[bibr18-13623613241292172] Hazen N. L. Black B. (1989). Preschool peer communication skills: The role of social status and interaction context. Child Development, 60(4), 867–876. 10.2307/1131028 2667904

[bibr19-13623613241292172] Heasman B. Gillespie A. (2019). Neurodivergent intersubjectivity: Distinctive features of how autistic people create shared understanding. Autism, 23(4), 910–921. 10.1177/1362361318785172 30073872 PMC6512057

[bibr20-13623613241292172] Heeman P. A. Lunsford R. Selfridge E. Black L. van Santen J. (2010). Autism and interactional aspects of dialogue. In Proceedings of the SIGDIAL 2010 Conference, 249–252.

[bibr21-13623613241292172] Hymas R. Badcock J. C. Milne E. (2022). Loneliness in autism and its association with anxiety and depression: A systematic review with meta-analyses. Review Journal of Autism and Developmental Disorders, 11, 121–156. 10.1007/s40489-022-00330-w

[bibr22-13623613241292172] Johnson S. A. Filliter J. H. Murphy R. R. (2009). Discrepancies between self- and parent-perceptions of autistic traits and empathy in high functioning children and adolescents on the autism spectrum. Journal of Autism and Developmental Disorders, 39, 1706–1714. 10.1007/s10803-009-0809-1 19626433

[bibr23-13623613241292172] Kent County Council. (2023). Schools with specialist resource provision.

[bibr24-13623613241292172] Kjelgaard M. Tager-Flusberg H. (2001). An investigation of language impairment in autism: Implications for genetic subgroups. Language and Cognitive Processes, 16(2–3), 287–308. 10.1080/01690960042000058 16703115 PMC1460015

[bibr25-13623613241292172] Lockton E. Adams C. Collins A. (2016). Do children with social communication disorder have explicit knowledge of pragmatic rules they break? A comparison of conversational pragmatic ability and metapragmatic awareness. International Journal of Language and Communication Disorders, 51(5), 508–517. 10.1111/1460-6984.12227 26916221

[bibr26-13623613241292172] Lord C. Rutter M. DiLavore P. S. Risi S. Gotham K. Bishop S. L. (2012). Autism diagnostic observation schedule, second edition (ADOS-2). Western Psychological Services.

[bibr27-13623613241292172] Loucas T. Charman T. Pickles A. Simonoff E. Chandler S. Meldrum D. Baird G. (2008). Autistic symptomatology and language ability in autism spectrum disorder and specific language impairment. Journal of Child Psychology and Psychiatry, 49(11), 1184–1192. 10.1111/j.1469-7610.2008.01951.x 19017030

[bibr28-13623613241292172] Maïano C. Normand C. L. Salva M. C. Moullec G. Aimé A. (2015). Prevalence of school bullying among youth with autism spectrum disorders: A systematic review and meta-analysis. Autism Research, 9(6), 601–615. 10.1002/aur.1568 26451871

[bibr29-13623613241292172] McKernan E. P. Kumar M. Di Martino A. Shulman L. Kolevzon A. Lord C. Narayanan S. Kim S. H. (2022). Intra-topic latency as an automated behavioral marker of treatment response in autism spectrum disorder. Scientific Reports, 12(3255), 1–10. 10.1038/s41598-022-07299-w 35228613 PMC8885715

[bibr30-13623613241292172] Ministry of Housing, Communities & Local Government. (2019). English indices of deprivation 2019. https://www.gov.uk/government/statistics/english-indices-of-deprivation-2019

[bibr31-13623613241292172] Mitchell P. Sheppard E. Cassidy S. (2021). Autism and the double empathy problem: Implications for development and mental health. British Journal of Developmental Psychology, 39(1), 1–18. 10.1111/bjdp.12350 33393101

[bibr32-13623613241292172] Nadig A. Less I. Singh L. Bosshart K. Ozonoff S. (2010). How does the topic of conversation affect verbal exchange and eye gaze? A comparison between typical development and high-functioning autism. Neuropsychologia, 48(9), 2730–2739. 10.1016/j.neuropsychologia.2010.05.020 20493890 PMC2935904

[bibr33-13623613241292172] Nguyen V. Versyp O. Cox C. Fusaroli R. (2022). A systematic review and Bayesian meta-analysis of the development of turn taking in adult–child vocal interactions. Child Development, 93(4), 1181–1200. 10.1111/cdev.13754 35305028 PMC9271548

[bibr34-13623613241292172] Nielsen M. Haun D. Kärtner J. Legare C. H. (2017). The persistent sampling bias in developmental psychology: A call to action. Journal of Experimental Child Psychology, 162, 31–38. 10.1016/j.jecp.2017.04.017 28575664 PMC10675994

[bibr35-13623613241292172] Ochi K. Ono N. Owada K. Kojima M. Kuroda M. Sagayama S. Yamasue H. (2019). Quantification of speech and synchrony in the conversation of adults with autism spectrum disorder. PLOS ONE, 14(12), Article e0225377. 10.1371/journal.pone.0225377 PMC6894781 31805131

[bibr36-13623613241292172] Pagmar D. Abbot-Smither K. Matthews D. (2022). Predictors of children’s conversational contingency. Language Development Research, 2(1), 139–179. 10.34842/2022-511

[bibr37-13623613241292172] Parish-Morris J. Liberman M. Ryant N. Cieri C. Bateman L. Ferguson E. Schultz R. T. (2016). Exploring autism spectrum disorders using HLT. In Proceedings of the Third Workshop on Computational Linguistics and Clinical Psychology, 74–84. 10.18653/v1/w16-0308 PMC7558465 33071446

[bibr38-13623613241292172] Paul R. Orlovski S. M. Marcinko H. C. Volkmar F. (2009). Conversational behaviours in youth with high-functioning ASD and asperger syndrome. Journal of Autism and Developmental Disorders, 39, 115–125. 10.1007/s10803-008-0607-1 18607708 PMC2819316

[bibr39-13623613241292172] Place K. S. Becker J. A. (1991). The influence of pragmatic competence on the likeability of grade-school children. Discourse Processes, 14(2), 227–241. 10.1080/01638539109544783

[bibr40-13623613241292172] Rifai O. M. Fletcher-Watson S. Jiménez-Sánchez L. Crompton C. J. (2022). Investigating markers of rapport in autistic and nonautisic interactions. Autism in Adulthood, 4(1), 3–11. 10.1089/aut.2021.0017 36600904 PMC8992924

[bibr41-13623613241292172] Sasson N. Faso D. Nugent J. Lovell S. Kennedy D. Grossman R. (2017). Neurotypical peers are less willing to interact with those with autism based on thin slice judgments. Scientific Reports, 7(40700). 10.1038/srep40700 PMC5286449 28145411

[bibr42-13623613241292172] Sperber D. Wilson D. (1995). Relevance: Communication and cognition (2nd ed.). Blackwell Publishing.

[bibr43-13623613241292172] Stagg S. D. Slavny R. Hand C. Cardoso A. Smith P. (2014). Does facial expressivity count? How typically developing children respond initially to children with autism. Autism, 18(6), 704–711. 10.1177/1362361313492392 24121180

[bibr44-13623613241292172] Stagg S. D. Thompson-Robertson L. Morgan C. (2022). Primary school children rate children with autism negatively on looks, speech and speech content. British Journal of Developmental Psychology, 41(1), 37–49. 10.1111/bjdp.12430 36003025

[bibr45-13623613241292172] Stivers T. Enfield N. J. Brown P. Englert C. Hayashi M. Heinemann T. Hoymann G. Rossano F. de Ruiter J. P. Yoon K. E. Levinson S. C. (2009). Universals and cultural variation in turn-taking in conversation. Proceedings of the National Academy of Sciences of the United States of America, 106(26), 10587–10592. 10.1073/pnas.0903616106 19553212 PMC2705608

[bibr46-13623613241292172] Straus J. N. (2013). Autism as culture. In Davis L. J. (Ed.), The disability studies reader (pp. 460–484). Taylor & Francis. https://www.taylorfrancis.com/chapters/mono/10.4324/9780203077887-45/autism-culture-lennard-davis

[bibr47-13623613241292172] Sturrock A. Chilton H. Foy K. Freed J. Adams C. (2022). In their own words: The impact of subtle language and communication difficulties as described by autistic girls and boys without intellectual disability. Autism, 269(2), 332–345. 10.1177/13623613211002047 PMC8814951 34291667

[bibr48-13623613241292172] Tager-Flusberg H. Anderson M. (1991). The development of contingent discourse ability in autistic children. Journal of Child Psychology & Psychiatry & Allied Disciplines, 32(7), 1123–1134. 10.1111/j.1469-7610.1991.tb00353.x 1838537

[bibr49-13623613241292172] van Steensel F. J. A. Bögels S. M. Perrin S . (2011). Anxiety disorders in children and adolescents with autistic spectrum disorders: A meta-analysis. Clinical Child and Family Psychology Review, 14, 302–317. 10.1007/s10567-011-0097-0 21735077 PMC3162631

[bibr50-13623613241292172] Wargo Aikins J. Collibee C. Cunningham J . (2017). Gossiping to the top: Observed differences in popular adolescents’ gossip. Journal of Early Adolescence, 37(5), 642–661. 10.1177/0272431615617291

[bibr51-13623613241292172] Warlaumont A. S. Oller D. Dale R. Richards J. A. Gilkerson J. Xu D. (2010). Vocal interaction dynamics of children with and without autism. Proceedings of the Annual Meeting of the Cognitive Science Society, 32.

[bibr52-13623613241292172] Wechsler D. (2011). Wechsler abbreviated scale of intelligence–second edition (WASI-II). NCS Pearson Inc.

[bibr53-13623613241292172] Wiig E. H. Secord W. A. Semel E. (2006). Clinical Evaluation of Language Fundamentals-fourth edition (CELF-4). NCS Pearson Inc.

[bibr54-13623613241292172] Williams D. (2010). Theory of own mind in autism: Evidence of a specific deficit in self-awareness? Autism, 14(5), 474–494. 10.1177/1362361310366314 20926458

[bibr55-13623613241292172] Ying Sng C. Carter M. Stephenson J . (2018). A systematic review of the comparative pragmatic differences in conversational skills of individuals with autism. Autism & Developmental Language Impairments, 3. 10.1177/2396941518803806

[bibr56-13623613241292172] Ying Sng C. Carter M. Stephenson J. Sweller N . (2020). Partner perceptions of conversations with individuals with autism spectrum disorder. Journal of Autism and Developmental Disorders, 50, 1182–1197. 10.1007/s10803-019-04348-8 31894461

