# Supplementary material for: Seeing it in others versus doing it yourself: Social desirability judgements and conversation production data from autistic and non-autistic children
Source: Autism. 2024 Nov 4;29(4):975–87. doi: 10.1177/13623613241292172 (PMC11967101; doi:10.1177/13623613241292172)
Supplement: sj-docx-1-aut-10.1177_13623613241292172 – Supplemental material for Seeing it in others versus doing it yourself: Social desirability judgements and conversation production data from autistic and non-autistic children [file sj-docx-1-aut-10.1177_13623613241292172.docx]

**Supplementary Materials**

**S1. Conversation Vignettes Conversation 1: Football**

**On-topic:**

1: I played football at the weekend, and my team scored three goals.

2: I love football, but I never really score any goals because I’m a defender. 1: My favourite football player is Harry Maguire and he’s a defender.

2: I remember watching him during the Euros tournament.

1: Yeah, I watched all of the Euros games with my dad and my brother. 2: Me too; we even got to go see one of the games at Wembley.

# Off-topic:

1: I played football at the weekend, and my team scored three goals. 2: I’ve noticed that it’s started to get dark really early.

1: Yeah, it’s sad that summer is officially over now. 2: I’ve just realised this new jumper has a hole in it.

1: Oh, you should definitely return it to the shop since you’ve only just bought it. 2: I think it might be lunchtime.

# Conversation 2: Guitar On-topic:

1: I had my first guitar lesson today.

2: I’ve always wanted to learn to play an instrument, but it seems really hard. 1: Yeah, there’s a lot to remember, but I think I could be pretty good at it.

2: Wow, maybe you could play guitar as your job in the future. 1: I really like the idea of being in a band when I’m older.

2: That’s so cool, you could be the lead guitarist in a rock band.

# Off-topic:

1: I had my first guitar lesson today.

2: I think I’m going to get a chicken wrap for lunch today. 1: I’m in the mood for pizza or maybe some pasta.

2: I can’t wait to find out what roles we’ve been given in the school play. 1: Me too; I really want to play Peter Pan.

2: I’ve been thinking about buying some new headphones for my computer.

# Conversation 3: Superheroes On-topic:

1: I think my favourite superhero is either Spiderman or Captain America. 2: They’re awesome, but my favourite has to be Iron Man.

1: He’s cool because he gets to fly around in his suits.

2: I think if I could have any superpower, I’d choose invisibility. 1: I quite like the idea of being able to teleport.

2: That would be good because then you’d never be late to anything!

# Off-topic:

1: I think my favourite superhero is either Spiderman or Captain America. 2: I love your new bag by the way; it’s a really nice colour.

1: Thank you, I bought it when I went to the town last week. 2: I think we should go for a walk at break-time.

1: That’s a good idea, I could do with some fresh air.

2: I can’t wait to go home later so I can start reading my new book.

# Conversation 4: Holiday

**On-topic:**

1: My family are going to book a holiday soon, but we can’t decide where to go. 2: I like going to hot countries, like Spain or Portugal.

1: Me too, I always go to the beach when I’m on holiday. 2: Me and my sister like going swimming in the ocean.

1: You have to be careful that you don’t get sunburnt though, because it can get really hot. 2: Yeah, once I got a really bad sunburn on the back of my neck.

# Off-topic:

1: My family are going to book a holiday soon, but we can’t decide where to go. 2: I really need to buy some new trainers; these ones are so scuffed.

1: Maybe you could look in the Nike shop for some new ones. 2: I heard that Jane is moving to a different town.

1: She’s going to be living closer to the seaside which will be fun. 2: I think it’s about to start raining.

# Conversation 5: Restaurant On-topic:

1: I went to that new restaurant in town last night.

2: I walked past it the other day and I thought it looked great.

1: Yeah, the food was really nice, and I tried sushi for the first time. 2: Wow, I’ve never had sushi before.

1: The only problem was that we had to wait ages for the food to come. 2: Oh, I guess they were really busy.

# Off-topic:

1: I went to that new restaurant in town last night. 2: Oh no, I think my library books are due in today.

1: Don’t worry, we can always go to the library on the way home. 2: I slept really badly last night because it was so hot.

1: Me too, I had to have my fan on all night, but it didn’t really help. 2: I need to throw this bubble-gum away; it’s lost all of its taste.

# Conversation 6: Friends On-topic:

1: I’m really looking forward to Sophie’s sleepover on Saturday.

2: Me too, I’m going to buy some sweets for us to have when we’re there. 1: I’ve already got some popcorn for when we watch films.

2: That’s great, but I really hope we don’t end up watching a horror film. 1: Yeah, I’d rather watch a funny animated film like Despicable Me.

2: Yeah, I love the minions!

# Off-topic:

1: I’m really looking forward to Sophie’s sleepover on Saturday. 2: I had a dream last night that I was flying an aeroplane.

1: That’s so cool; it would be awesome if you could do that in real life. 2: My shoulder’s really been hurting since our PE lesson.

1: Oh no, maybe you’ve pulled a muscle or something. 2: I think I might be allergic to my new shampoo.

# S2. Comic-strip-style Graphics

**On-topic:**

**Conversation 1: Football**


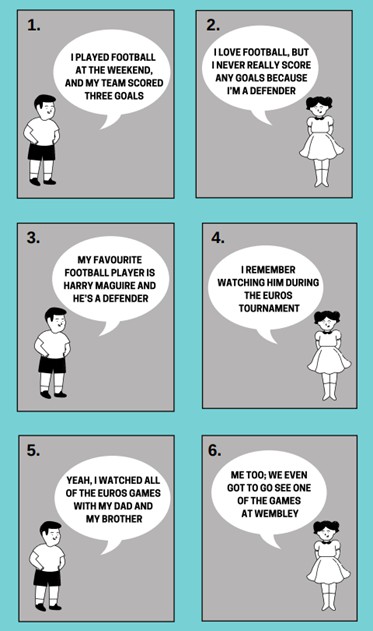


**Off-topic:**


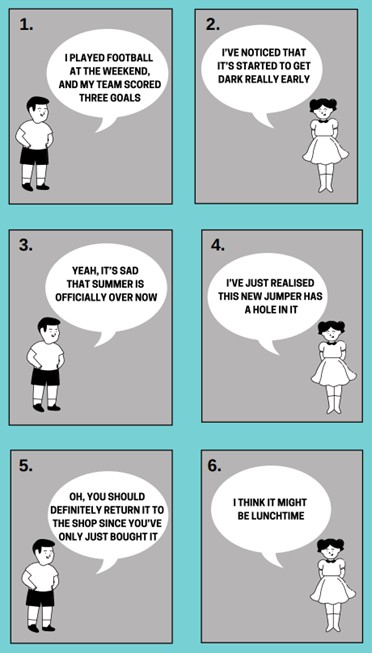


**Conversation 2: Guitar**

**On-topic:**


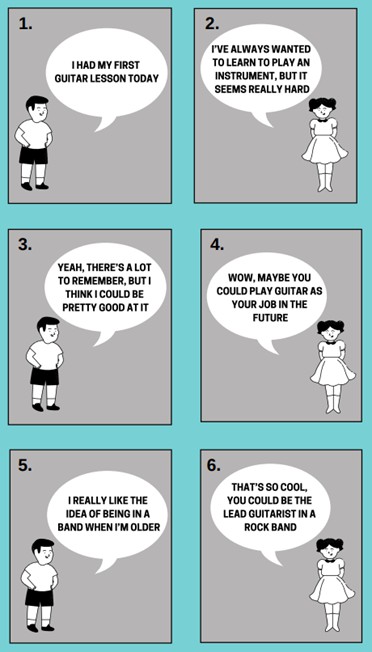


**Off-topic:**


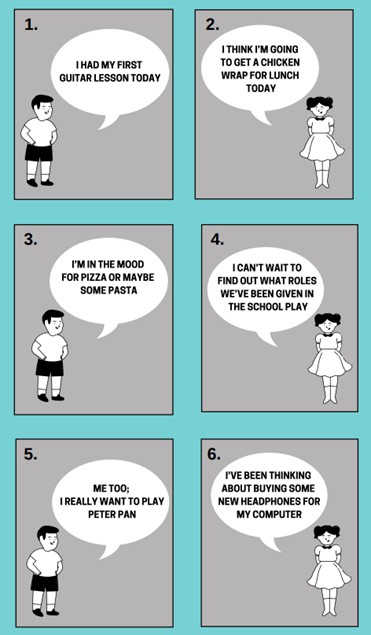


**Conversation 3: Superheroes**

**On-topic:**


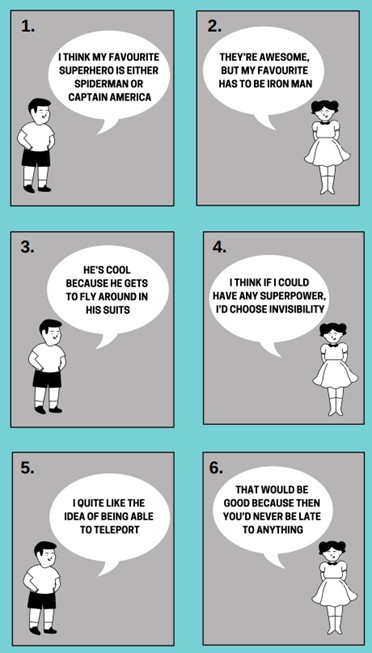


**Off-topic:**


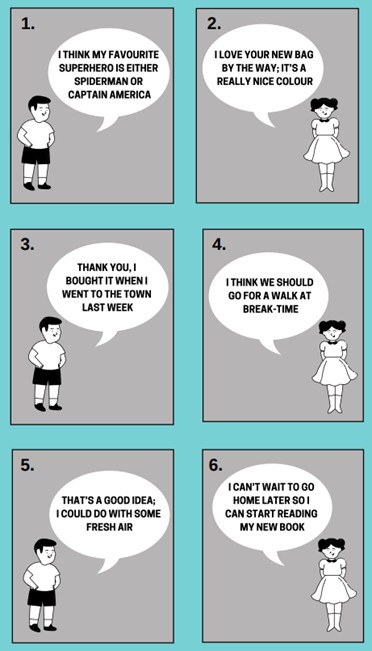


**Conversation 4: Holiday**

**On-topic:**


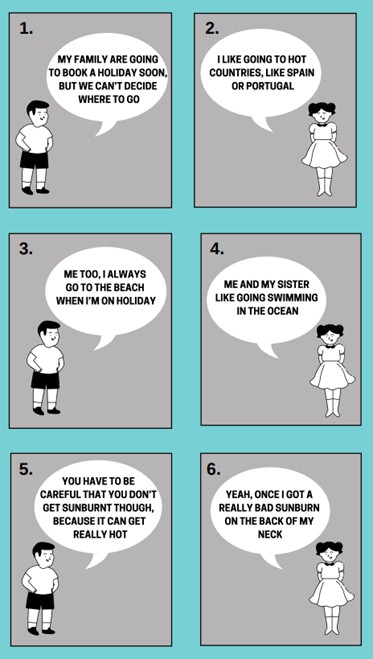


**Off-topic:**


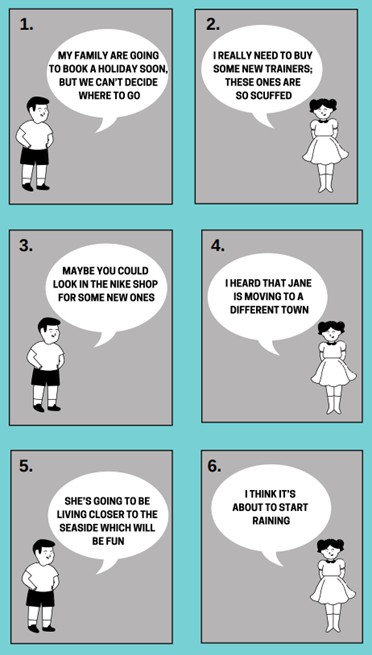


**Conversation 5: Restaurant**

**On-topic:**


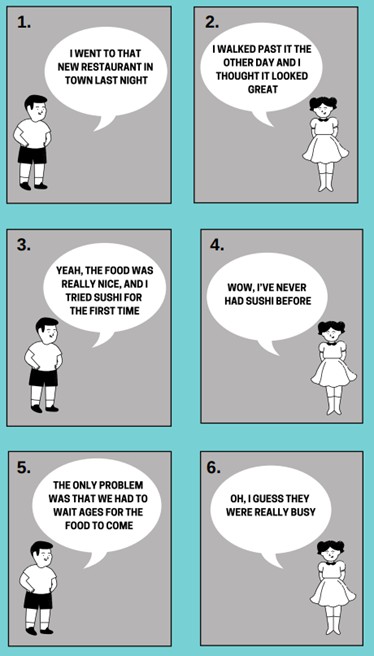


**Off-topic:**


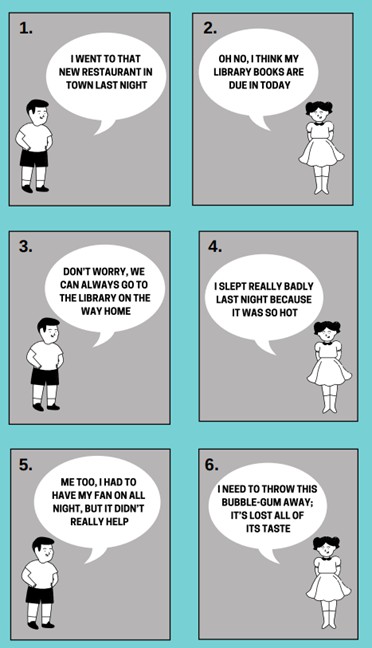


**Conversation 6: Friends**

**On-topic:**


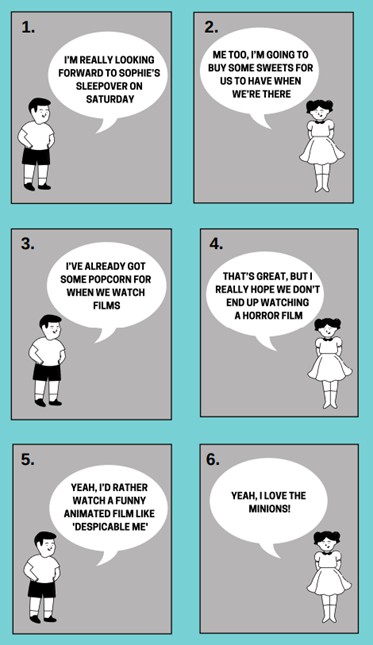


**Off-topic:**


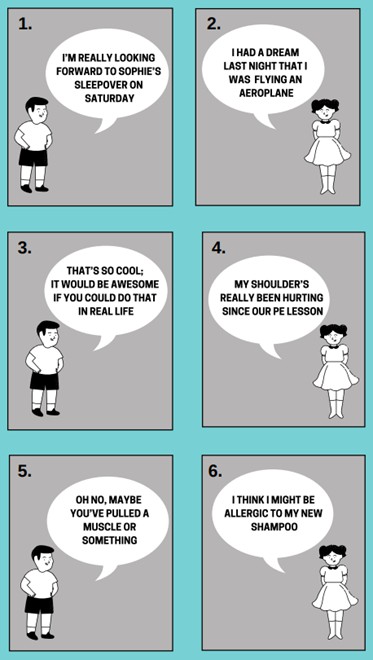


**S3. Presentation Order**

**Initial Tasks:**

| Task | Details |
| --- | --- |
| **Volume Check** | This task ensured that the volume on participants’ devices was set to an appropriate level. Participants listened to a series of non-verbal tones and were asked to indicate which tone had a higher pitch. |
| **Comprehension Task** | Participants listened to a 30-second story about a boy who had lost his dog. This was the same length as the conversation stimuli (six- utterances). Participants then answered 4 multiple-choice comprehension questions, none of which required inferences e.g., “What went missing?” and “What had Noah’s puppy been doing?”.  Participants were required to answer at least 75% of the questions correctly to be included in the study. |
| **Judgment Task Familiarity** | Before being presented with the main experimental trials, participants were taught how to use the sliders by rating two unrelated statements e.g., “I enjoy going to school”. This also allowed the experimenter to chat briefly with the child before commencing the main session.  Following the task instructions, participants also completed two practice items which contained basic, two-utterance conversations. |

**Experimental Tasks:**

Session 1:

1. Vignette block 1 (Test conversations 1-3)
2. Conversation Probes 1-3
3. Referential Communication Task block 1
4. Conversation Probes 4-6
5. Non-verbal Reasoning Test (WASI-matrices)
6. Vignette block 2 (Test conversations 4-6)
7. Conversation Probes 7-9

Session 2:

1. Referential Communication Task block 2
2. Vignette block 3 (Test conversations 7-9)
3. Conversation Probes 10-12
4. Language Ability Test (CELF-4 Recalling Sentences)
5. Vignette Control items
6. Referential Communication Task block 3

# S4. Conversation Probes

**Prompt: Video of dog dancing**

- I had a dog like that when I was little, but he was a bit naughty.
- He used to steal socks from the laundry.
- Some people prefer rabbits as pets because they are easier to look after.

# Prompt: Duck on screen for Referential Communication Activity

- Me and my family like going to the park to feed the ducks.
- Sometimes we have a picnic, and the ducks try to steal our food.
- There’s a lake in our park where you can go in boats.

# Prompt: Video of a man slipping on ice

- Once it was so icy that our car slid across the road.
- Winter can be fun when it snows.
- We had a snowball fight in the garden last year.

# Prompt: Video of a cat being made jump

- My sister is scared of cats because one scratched her once.
- My grandma’s cat brings mice into the house.
- I used to want to be a vet, so I’m not scared of any animals.

# Prompt: Video of tired dog

- I had to wake up really early this morning and I found it so difficult.
- I was probably tired because I played Minecraft until 9 o’clock.
- I like computer games that you can play with your friends.

# S5. Coding Schedule for Responses to Conversation Probes

1. **Contingent responses**

Our variable ‘contingent responses’ was the sum of ‘contingent statements’ and ‘contingent questions’.

# . Contingent Statements

Child multi-word declarative utterances that are contingent on E’s utterance; that is, they provide an **appropriate, informative, on-topic** response which **adds information** to the experimenter’s prior utterance, **thus providing the experimenter with an opportunity to follow in on the child’s utterance.** This can be about the specific experience mentioned in E’s turn, as in:

E: But when I tried to copy it I couldn't find a yellow pencil anywhere C: You could have used a yellow crayon.

In other examples of other-contingent utterances, children may bring in their own experience in ways that are relevant to the specific topic of E’s turn as in:

E: I’m going on holiday next week C: I’m going on holiday in July

Utterances are not contingent if they refer to an earlier topic in the conversation. They are also not contingent if they only relate in a very generic way to the topic (e.g., Minecraft, tennis) without being about the specific sub-topic of E’s turn.

# 1.2. Contingent Questions

Any multi-word contingent response that takes a question form.

**NB**:

- Non-contingent questions (that switch the topic) are just coded as ‘non-contingent’ (see below).
- One-word questions (e.g. ‘What?’) are coded under ‘Minimal’ responses (see below).

# Non-contingent responses

Utterances that do not maintain the topic of the immediately preceding utterance of the conversation partner. These can either be declarative or interrogative. For example:

E: I’m going on holiday next week C: I’m having pizza for dinner

# Returns to Previous Topic

Even if the utterance refers to a previous topic of conversation, this is coded as non- contingent as it does not directly relate to a previous turn. An example of this is:

E: Oh, I hate spiders.

E: But yes, I haven’t built the whole lego town, but I have helped my brother do it.

P: I’ve been slain by one because they are so strong.

*Here, P is returning to the topic of spiders even though E has moved on to talking about the lego town*.

# Not on Specific Topic

Utterances which do not refer to the specific topic of E’s immediately preceding conversation turn, but only to the general topic under discussion (Minecraft, tennis) are non-contingent. An example of this is:

E: [*talking about specific aspect of Minecraft*] so I’ve died a few times falling in myself but it’s quite good at keeping baddies out, is lava

P: mm cause Minecraft is basically made of squares.

# Tangentially Related Utterances

Sometimes non-contingent responses are related tangentially e.g.,

E: and I baked these with my mum because she’s a very good baker P: My mum can speak three languages.

In this example, the participant picks up on the ‘mum’ element and mentions information relating to that, rather than to the topic of baking.

# Utterances with Unclear Content

Utterances that are initially unclear but then become clear later in a separate utterance would still be counted as non-contingent as they do not make sense at the time. For example:

E: I have a dog

P: Yeah, Biffy loves going out in the rain

It may later become apparent that Biffy is the name of the child’s dog, but sine this is not clear at the time the utterance would be coded as non-contingent.

# Bizarre Utterances

Bizarre utterances (e.g., ‘Apple jumped up and bumped his head’) or utterances which feel as if they have been rote-learning from somewhere because they are overly formal or weird in context (e.g., ‘my teacher has a very good reputation’) are always coded as ‘non-contingent’ as the relevance to the context cannot be determined.

# Environmental Switches

Non-contingent responses may involve the child switching away from the current topic to talk about an object they can see in the immediate environment (e.g., ‘Look, at my new toy’) or asking to do something else (e.g., ‘Can we play another game?’).

# Interruptions

If the child interrupts the experimenter so that the experimenter makes a substantial start but does not finish his / her turn, this is coded as non-contingent.

# One-word Off-topic Utterances

These include one-word utterances which are clearly off the topic of the immediately preceding utterance e.g.,:

E: We like to have picnics. P: Minecraft!

**Rule of thumb:**

When in doubt about a contingent/non-contingent judgment, be conservative and opt for non- contingent. If you struggle to decide how to code the response, it is not contingent enough to allow for the normal flow of conversation.

# Minimal Responses

Minimal responses are those which are not off-topic, but which **do not add information** to the preceding conversational turn and thus they **do not provide the conversation partner with an opportunity to ‘follow in’.**

# Appropriate minimal responses

The following types of minimal response are counted as ‘appropriate’ because the content is clearly specific to the immediately preceding turn and thus these utterances clearly indicate that the child is engaging with the content.

- - 1. One-word contingent utterances with **semantic** content which is **relevant** or **contingent** to the experimenter’s immediately preceding turn. (Included here is the ‘additional’ word, i.e., a definite article ‘the’ or indefinite article ‘a’).

Examples include:

- - - - - What?
      - - Where?
      - - Why?
      - - Doggy!
      - - Pussy cat
      - - Probably
      - - The googles
      - - Neither
      - - Our one
      - - This
      - - Everywhere
      - - Fun!
      - - No!

**NB**: If the one-word utterance is non-contingent (e.g. if the child shouts ‘Minecraft’ on seeing a picture of Minecraft), these are instead coded as ‘non-contingent’).

- - 1. Two-word or three-word phrases with semantic content but which do not easily allow the experimenter to ‘follow in’ with something that has content.

Examples include:

- - - - Me too
      - I don’t
      - That’s nice
      - That’s cool
      - That’s right
      - Silly old you.

**3.1.2** Listening phrases which provide an **affective** comment on what the speaker is saying, but do not provide the conversation partner with an opportunity to then follow in on them.

Examples include:

- Did you?
- You did?
- Did he?
- Have you?
- Is it?
- Does it?
- Really?
- Oh no!
- Wow!
- No way!
- Urgh
- Ewwww
- aww

# Inappropriate minimal responses

The following types of minimal response are counted as ‘inappropriate’ because the content is generic and thus the child could plausibly have produced this because s/he knows it is his / her turn to talk but the response does not necessarily indicate that the child has processed what the experimenter has just said.

- - 1. One-word utterances **empty of semantic content** (e.g., “okay”) or vocalisations which are **verbal but not actually words** (e.g., “Mhmm”)

Examples include:

- - - - oh
      - right
      - yeah
      - yep
      - Okay
      - Oh okay
      - Uh okay
      - mm
      - uh-huh
      - Ohh

**3.2.2. Potential echolalia,** including any direct **repetitions** of the experimenter e.g., E: I went to beach in summer

C: I went to the beach in summer

# 3.2.3. Stimming-type noises

Examples include humming, clicking with mouth, repeated noises (e.g., badadday, baddaday)

# Minimal Other

Minimal responses for which it is unclear whether they should be coded as an ‘appropriate’ versus an ‘inappropriate’ response.

# Non-verbal / Null responses

To code child verbal silence as a null response, the experimenter needed to have left at least a 3000 ms pause between the offset of one utterance and the onset of the next. Based on the video-recordings, null responses are categorised into the following sub-types:

# Appropriate Non-verbal Responses

The following types of non-verbal response are counted as ‘appropriate’ because they indicate a clear reaction to probe.

# Emotive facial expressions

Examples include raising eyebrows, widening eyes, dropping open mouth, wincing etc.

# 5.1.2 Gestures or expressions demonstrating understanding

Examples include smiling or nodding or both.

# Inappropriate non-verbal responses

The following types of non-verbal response are counted as ‘inappropriate’ because they do not indicate clear response to the probe.

# Gestures or expressions demonstrating a lack of engagement

Examples include looking away or shoulder shrugs.

# Completely null responses

Looking at the experimenter with no clear response to the experimenter’s turn e.g., just staring at the screen.

# NB:

- - - - If the child produces a verbal response after a long (e.g. as long as or longer than a 2000 msec pause) before the experimenter talks again, then this is coded in terms of the verbal content and not as a null response.
      - If the child produced both a verbal and non-verbal response (e.g., [Smiles] “Yeah”), the verbal response would be coded. Non-verbal responses are only coded int eh absence of a verbal response.

# Excluded Responses

- 1. If a participant laughed in response the probe, this response was coded as ‘N/A’. This is because it is difficult to establish the appropriacy of laughter in relation to the probes.
  2. We do not code child back-channels. These are ‘minimal-like’ utterances (e.g. ‘oh’, ‘right’, ‘gosh’, ‘wow’) produced while the experimenter is still speaking in order to indicate active listening.

# S6. Coding Schedule for Verbal Justifications of Ratings

**Topic:**

# Mentions of Topic may include:

- - - Off-topic, random, inconsistent, unrelated, irrelevant, opposite
    - “She changes/switches/interrupts the [direction of] the conversation/topic/subject”
    - “She’s not talking about the same thing”
    - “She didn’t say anything linked/connected/to do with what the boy said”
    - “She didn’t respond to what the boy was saying”
    - “She said something else/different to what the boy was talking about”
    - “She didn't follow up”
    - “She said/jumped to a new thing”
    - “She doesn’t focus on the same subject”
    - “She’s not responding/replying to the boy’s point”
    - “Making up another conversation/topic”

# Active Listening Comments:

- - - “Ignoring/interrupting the boy / what he’s saying”
    - “Seems distracted
    - “Not focussing”
    - “Isn’t listening to the boy”
    - “Isn’t paying attention to the boy”
    - “Not engaged in the conversation”
    - “Not taking part”
    - “Doesn’t care what the boy is saying”
    - “Doesn’t seem interested” / “Doesn’t take an interest”

# Difficulties Comments:

- - - “She might be thinking of / didn’t know what to say”
    - “She might struggling be to speak”
    - “Short attention span”
    - “She might have a disability”
    - “She might be confused”

**Timing:**

# Mentions of Timing may include:

- - - Delayed, pauses, waits, hesitates, stalls
    - “Slow / late responses”
    - “Leaves gaps”
    - “Waiting too long to say something”
    - “Takes a long time to respond” / “Took a while to speak”
    - “The boy is waiting for her to say something”
    - “Taking her time”
    - “The conversation doesn’t flow”

# Active Listening Comments:

- - - “Ignoring the boy / what he’s saying”
    - “Seems distracted”
    - "(Not) focussing / interested / involved”
    - “Is (not) listening to the boy”
    - “Is (not) paying attention to the boy” / “Is (not) concentrating”
    - “(Dis)engaged in the conversation”
    - “Not interrupting” / “acknowledging the boy’s replies”

# Difficulties Comments:

- - - “She might be thinking of / can’t remember what to say”
    - “She might struggling be to speak” / “having trouble speaking”
    - “She might have a disability”
    - “She might be confused”
    - “She’s trying to make sense of what he said”
    - “She might be stammering”

# S7. Judgement x Production Correlations

**Whole sample:**

|  | Mean proportion of contingent responses | Mean proportion of non- contingent responses |
| --- | --- | --- |
| Mean ratings of On-topic vignettes | *r*s = -.08, *p* = .490 | *r*s = -.10, *p* = .400 |
| Mean ratings of Off-topic vignettes | ***r*s = -.24, *p* < .05** | *r*s = -.09, *p* = .460 |
| Mean difference in Topic ratings | *r*s = .16, *p* = .174 | *r*s = .03, *p* = .790 |
|  |  |  |
|  | Mean response latency |  |
| Mean ratings of Typical vignettes | *r*s = .08, *p* = .565 |  |
| Mean ratings of Delayed vignettes | *r*s = .06, *p* = .657 |  |
| Mean difference in Timing ratings | *r*s = -.02, *p* = .886 |  |
| **Autistic group:** |  |  |
|  | Mean proportion of contingent responses | Mean proportion of non- contingent responses |
| Mean ratings of On-topic vignettes | *r*s = -.23, *p* = .149 | *r*s = -.16, *p* = .361 |
| Mean ratings of Off-topic vignettes | *r*s = -.18, *p* = .293 | *r*s = -.16, *p* = .353 |
| Mean difference in Topic ratings | *r*s = .04, *p* = .833 | *r*s = .09, *p* = .597 |
|  |  |  |
|  | Mean response latency |  |
| Mean ratings of Typical vignettes | *r*s = .02, *p* = .908 |  |
| Mean ratings of Delayed vignettes | *r*s = -.10, *p* = .591 |  |
| Mean difference in Timing ratings | *r*s = .06, *p* = .763 |  |
| **Non-autistic group:** |  |  |
|  | Mean proportion of | Mean proportion of non- |
|  | contingent responses | contingent responses |
| Mean ratings of On-topic vignettes | *r*s = .02, *p* = .890 | *r*s = .01, *p* = .973 |
| Mean ratings of Off-topic vignettes | *r*s = -.23, *p* = .176 | *r*s = -.00, *p* = .982 |
| Mean difference in Topic ratings | *r*s = .20, *p* = .239 | *r*s = .04, *p* = .804 |

Mean response latency Mean ratings of Typical vignettes *r*s = .24, *p* = .263 Mean ratings of Delayed vignettes *r*s = .29, *p* = .175 Mean difference in Timing ratings *r*s = .03, *p* = .905
